# DNA barcoding of *Culicoides* biting midges (Diptera: Ceratopogonidae) and detection of *Leishmania* and other trypanosomatids in southern Thailand

**DOI:** 10.1186/s13071-025-06812-0

**Published:** 2025-05-29

**Authors:** Piyapat Tepboonrueng, Thanapat Pataradool, Rungfar Boonserm, Luke W. Rimmer, Kanok Preativatanyou, Sakone Sunantaraporn, Padet Siriyasatien

**Affiliations:** 1 Medical Sciences Program, Faculty of Medicine, Chulalongkorn University, Bangkok, Thailand; 2 Department of Parasitology, Faculty of Medicine, Chulalongkorn University, Bangkok, Thailand; 3 Center of Excellence in Vector Biology and Vector-Borne Diseases, Department of Parasitology, Faculty of Medicine, Chulalongkorn University, Bangkok, Thailand; 4 Department of Biosciences, Faculty of Health and Life Sciences, University of Liverpool, Liverpool, UK

**Keywords:** *Culicoides* biting midges, *Leishmania martiniquensis*, *Leishmania orientalis*, *COI* gene, Thailand

## Abstract

**Background:**

Biting midges of the genus *Culicoides* play an important role in the transmission of pathogenic arboviruses and parasites. Thailand has documented more than 100 species of *Culicoides*; however, several cryptic species complexes remain to be clarified. Recent studies in areas with leishmaniasis indicate that several species of *Culicoides* might be potential vectors of *Leishmania* in the subgenus *Mundinia*, but evidence supporting the hypothesis is still lacking. Therefore, the diversity of *Culicoides* biting midges and their potential role as vectors of leishmaniasis in southern Thailand remains uncertain.

**Methods:**

Female *Culicoides* biting midges were collected using Centers for Disease Control and Prevention (CDC) ultraviolet (UV) light traps from four locations within leishmaniasis-affected areas in three provinces of southern Thailand, including Nakhon Si Thammarat, Krabi, and Surat Thani. *Culicoides* species were identified based on the morphology of wing spot patterns and subsequently confirmed by cytochrome c oxidase subunit I (*COI*) Sanger sequencing. A potential cryptic species was classified using an integrative taxonomic approach associated with DNA barcoding identification by Barcode of Life Database (BOLD) and Basic Local Alignment Search Tool (BLAST) searches. Furthermore, three different methods of species delimitation, namely ASAP [Assemble Species by Automatic Partitioning], TCS [Templeton, Crandall, and Sing], and PTP [Poisson Tree Processes], were employed to verify the sequences into the molecular operational taxonomic unit (MOTU). Detection of *Leishmania* and other trypanosomatid parasites was performed by polymerase chain reaction (PCR) based on the *ITS1* region and small subunit *SSU* ribosomal RNA (rRNA) gene, followed by Sanger sequencing and haplotype diversity analysis. The identification of host blood sources was carried out using host-specific multiplex PCR.

**Results:**

A total of 716 unfed midges and 159 blood-fed specimens were morphologically identified into 25 species belonging to five subgenera (*Avaritia*, *Hoffmania*, *Meijerehelea*, *Remmia*, and *Trithecoides*) and four species groups (*Clavipalpis*, *Ornatus*, *Shermani*, and *Shortti*). Two unidentified specimens were classified into two subgenera (*Trithecoides* and *Avaritia*). The DNA barcoding identification exhibited an 82.20% success rate. Species delimitation analyses demonstrated the presence of cryptic species complexes, categorized into six species: *Culicoides actoni*, *C. orientalis*, *C. huffi*, *C. palpifer*, *C. clavipalpis*, and *C. jacobsoni*. Furthermore, 6.42% of the *Culicoides* biting midges tested positive for *Leishmania* DNA in three sampling sites in Nakhon Si Thammarat and Surat Thani provinces (with no positive results in Krabi province). Furthermore, the sympatric infection of *Leishmania martiniquensis* and *Leishmania orientalis* was identified in several *Culicoides* species in Ron Phibun and Phunphin districts in Nakhon Si Thammarat and Surat Thani, respectively. In contrast, *L. orientalis* was detected in Sichon district, Nakhon Si Thammarat province. A genetic diversity analysis revealed high haplotype diversity and relatively low nucleotide diversity in both parasite populations. Additionally, *Crithidia* sp. and *Crithidia brevicula* were detected in *Culicoides peregrinus* and *Culicoides* subgenus *Trithecoides*. The analysis of the host blood meal from Ron Phibun also demonstrated that *Culicoides* had fed on cows, dogs, and chickens, and mixed blood preferences for humans and cows or chickens and cows were detected.

**Conclusions:**

The findings of the present study demonstrate the presence of mixed blood hosts and co-circulation of *L. martiniquensis* and *L. orientalis* in *Culicoides* in areas of leishmaniasis, as well as cryptic species of *Culicoides* biting midges, through an integrative taxonomic approach. These findings support the hypothesis that *Culicoides* biting midges may serve as potential vectors in southern Thailand, and vector diversity is a contributing factor to the risk of zoonotic transmission.

**Graphical Abstract:**

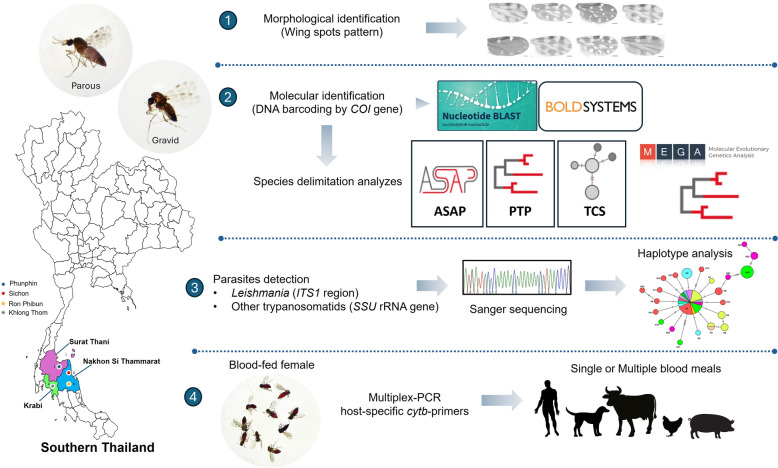

**Supplementary Information:**

The online version contains supplementary material available at 10.1186/s13071-025-06812-0.

## Background

Biting midges of the genus *Culicoides* are small, hematophagous insects belonging to the family Ceratopogonidae [1]. These flies are widespread and diverse, with approximately 1450 species recorded to date, of which 96% feed on avians and mammals [2]. It has been demonstrated that several species are associated with the transmission of arboviruses that impact both domestic animals and humans, including bluetongue virus (BTV), African horse sickness (AHSV), and Oropouche virus (OROV) [3, 4]. This insect has been incriminated as a vector of filarial nematodes [1], *Trypanosoma* [5], haemosporidian parasites [6], and most importantly, *Leishmania* [7–9].

Leishmaniasis is a vector-borne disease caused by obligate intracellular parasites of the genus *Leishmania* [10]. The World Health Organization (WHO) 2022 report indicates that 99 countries are endemic for leishmaniasis [11]. Of these, 71 countries have reported cases of both visceral leishmaniasis (VL) and cutaneous leishmaniasis (CL), while nine and 19 countries have reported cases of VL and CL, respectively [12, 13]. It is noteworthy that both VL and CL are endemic in Thailand, representing a significant public health challenge [14]. Recent studies and surveillance data indicate that the incidence of leishmaniasis in Thailand is a matter of growing concern due to the ongoing transmission of the disease [14]. To effectively interfere with disease transmission, a comprehensive understanding of the vectors and reservoirs that maintain the transmission cycles of pathogens is critical. In Thailand, the predominant species of *Leishmania* are *Leishmania martiniquensis* and *L. orientalis*, which have been previously reported in endemic areas of the northern and southern regions [15, 16]. Previous studies have reported on the subgenus *Mundinia* in traditional phlebotomine sand fly vectors of the genera *Sergentomyia* [17–19], *Phlebotomus* [19], and *Grassomyia* [20].

Recent studies have reported that biting midges (Diptera: Ceratopogonidae) have been identified as potential vectors of leishmaniasis in Thailand [21, 22], expanding the known range of vectors for this disease beyond sand flies. This discovery highlights the need for further research to elucidate the role of biting midges in the transmission dynamics of *Leishmania* parasites. Recent research findings from laboratory-based infection experiments noted that the *Leishmania* subgenus *Mundinia*, encompassing *L. chancei* (formerly referred to as “*Leishmania* sp. Ghana”), *L. martiniquensis*, and *L. orientalis*, possesses the capacity to metamorphose into metacyclic promastigotes within *Culicoides sonorensis*, and subsequently transmits to mice through the process of biting [9]. These findings suggest that the midges of the genus *Culicoides* may play a crucial role in the transmission of *Mundinia* species, in particular *L. martiniquensis* and *L. orientalis*. The first evidence of a molecular survey on *Leishmania* detection in biting midges from Thailand was conducted by Sunantaraporn et al., demonstrating the presence of *L. martiniquensis* DNA in *Culicoides mahasarakhamense* from Lamphun province of northern Thailand [21]. In southern Thailand, increasing cases of autochthonous leishmaniasis have been continuously reported [16, 22]. Studies have been conducted on the vectors of leishmaniasis in three locations; Songumpai et al. reported that *Culicoides peregrinus*, *C. oxystoma*, *C. mahasarakhamense*, *C. huffi*, *C. fordae*, and *C. fulvus* tested positive for *L. martiniquensis* DNA, while *L. orientalis* DNA has been reported in *C. peregrinus* and *C. oxystoma* from Songkhla Province [22]. In addition, Kaewmee et al. reported the detection of *L. martiniquensis* DNA in *C. peregrinus*, which was collected from Nakhon Si Thammarat province [23]. Conversely, no evidence of *Leishmania* was detected in *Culicoides* biting midges collected from Phatthalung province, as reported by Sunantaraporn et al. [24].

Previous studies in Thailand have focused on identifying *Culicoides* biting midges based on the morphology of their wing pigmentation patterns [25, 26]. Several studies have proposed the efficacy of DNA barcoding, utilizing the cytochrome c oxidase subunit I (*COI*) of the mitochondrial DNA, as a molecular marker for *Culicoides* species identification in different countries of Asia, including India [27], Japan [28], China [29], and Thailand [30–32]. Furthermore, an integrative taxonomic approach for species delimitation-associated DNA barcodes has been shown to potentially facilitate the identification of cryptic species complexes within the genus *Culicoides* [33]. Studies on *Culicoides* DNA barcoding in Thailand have been performed in several areas of animal sheds [30–32, 34]. Additionally, most studies on *Culicoides* composition in leishmaniasis areas were performed based on morphological characteristics [24, 35, 36]. However, only two studies on endemic areas for leishmaniasis have confirmed *Culicoides* species using *COI*-based identification in Thailand [21, 22]. Therefore, the present study aimed to investigate the DNA barcoding of *Culicoides* biting midges to demonstrate the species diversity or cryptic species complex, as well as the molecular detection of *Leishmania* parasites and other trypanosomatids in *Culicoides* biting midges collected from Nakhon Si Thammarat, Surat Thani, and Krabi provinces of southern Thailand. The findings of this study may be useful for monitoring and determining the vectorial capacity as well as the epidemiology of leishmaniasis and *Culicoides* biting midges in endemic areas in southern Thailand.

## Methods

### Sampling sites and biting midge collection

**Fig. 1:**
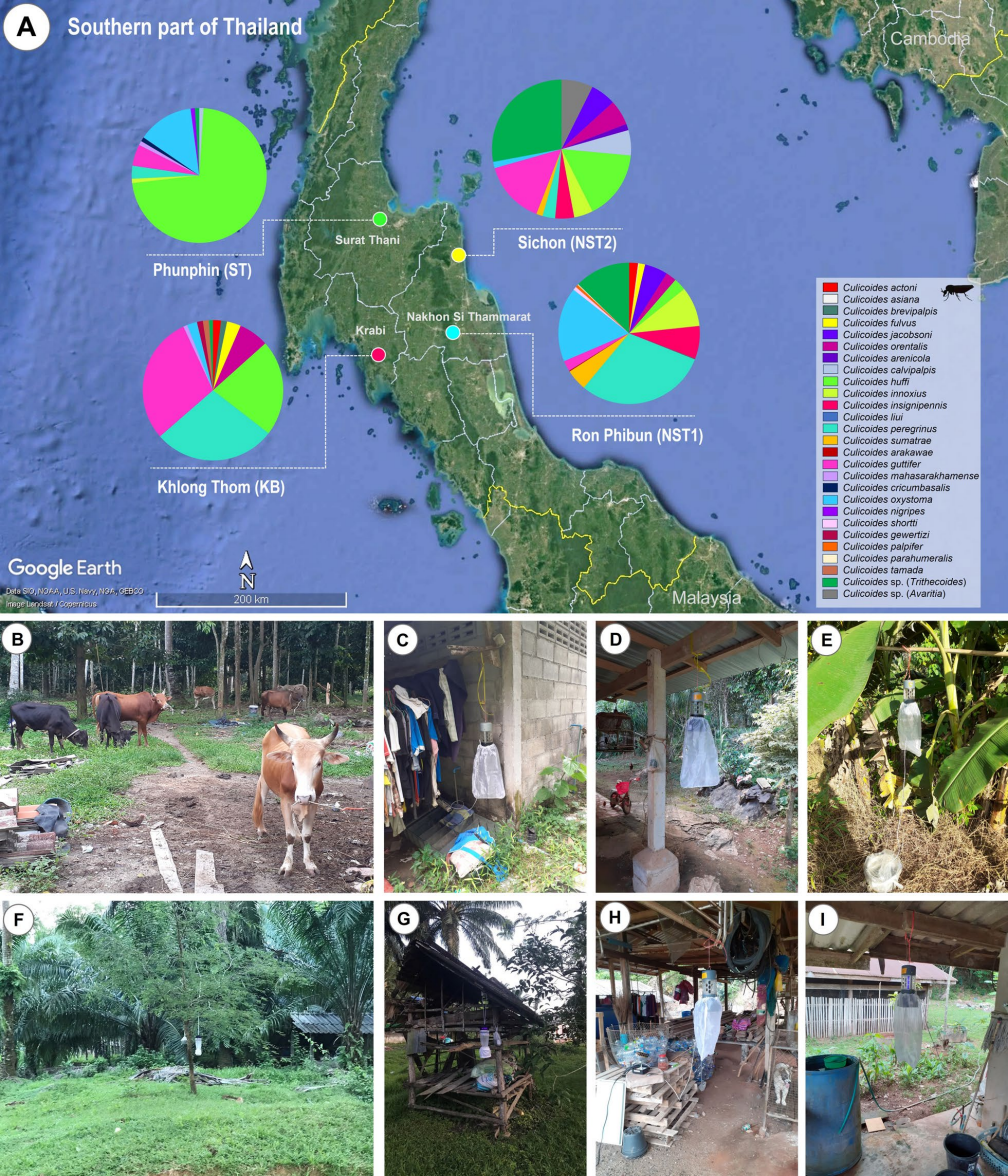
Map of the southern region of Thailand (**A**), demonstrating *Culicoides* species diversity and locations of trapping in four collection sites, including Nakhon Si Thammarat [NST1 (**B**, **C**) and NST2 (**D**, **E**)], Krabi (KB) (**F**, **G**), and Surat Thani (ST) (**H**, **I**) provinces. The map was obtained and modified from Google Earth Pro version 7.3.4.8248 (https://www.google.com/earth/about/)

*Culicoides* biting midges were collected from three provinces in southern Thailand, around houses of new symptomatic and asymptomatic leishmaniasis cases. The sampling sites are depicted in Fig. 1, and were located in Ron Phibun district (8°16′10.8″N 99°49′31.5″E) (NST1) and Sichon district (9°0′46″N 99°46′42″E) (NST2) from Nakhon Si Thammarat province; Khlong Thom district (8°2′54″N 99°12′50″E) (KB) from Krabi province; and Phunphin district (9°5′59″N 99°14′42″E) (ST) in Surat Thani province. Midge specimens were captured around the houses of people with a history of leishmaniasis, where domestic animals such as cows, dogs, and chickens were raised in the same neighborhood. The Centers for Disease Control and Prevention (CDC) light traps equipped with a 25 W ultraviolet (UV) light bulb were operated between 6:00 p.m. and 6:00 a.m. for three consecutive nights between 31 July and 3 August 2024. The positioning of the four traps was at an approximate height of 1.5 m above ground level, with the traps being situated near the cattle shed and chicken coop. Subsequently, insect collection bags were stored at −20 °C for 30 min to anesthetize the insects inside. All female *Culicoides* biting midges were morphologically sorted from other insects according to their wing spot patterns under a stereomicroscope (Olympus, Tokyo, Japan). Non-engorged and blood-engorged biting midges were placed separately in a 1.5 ml microcentrifuge tube containing 70% ethanol and kept in an ice box before being sent to the Center of Excellence in Vector Biology and Vector-Borne Diseases, Department of Parasitology, Faculty of Medicine, Chulalongkorn University. Non-engorged female (parous and gravid) biting midges were selected for screening of *Leishmania* parasites and other trypanosomatids, while blood-engorged biting midges were subjected to blood meal identification. All specimens were then taxonomically identified according to Wirth and Hubert (1989) [37] using characters visible on unmounted specimens.

### Nondestructive DNA extraction

For non-engorged specimens, genomic DNA (gDNA) was extracted from the whole body of individual *Culicoides* biting midges by digestion in 100 µl of cell lysis buffer (GeneAll, Seoul, Korea), treated with 10 µl of proteinase K solution, and incubated at 50 °C for 16 h. The lysate solution was further extracted following the manufacturer’s instructions using the Genti™ 32 automated nucleic acid extraction instrument based on a magnetic bead system (GeneAll, Seoul, Korea). The DNA quality and concentration were measured using the NanoDrop 2000c spectrophotometer (Thermo Fisher Scientific, Waltham, MA, USA). The extracted DNA was stored at −20 °C until the next processing. The remaining insect carcass was kept in a 1.5-ml microcentrifuge tube containing 70% ethanol solution in case retrospective morphological confirmation of identification was required. To detect host DNA in engorged midges, the abdomens of the engorged midges were removed, placed in individual 1.5-ml microcentrifuge tubes, and crushed using a sterilized plastic pestle. DNA extraction was performed as described above.

### Molecular identification of *Culicoides* species

The *COI* gene was targeted for species identification of *Culicoides* biting midges. A 523-base-pair (bp) fragment of the *COI* gene was amplified using C1-J-1718 and C1-N-2191 primers described previously [38]. If the amplification with the first primer pair failed, the primers LCO1490 and HCO2198 were used [39]. The PCR condition of the two primer sets was carried out as previously described by Mathieu et al. [40] and Harrup et al. [27]. For confirmation of the *Culicoides* species, the PCR products were purified using the FavorPrep™ GEL/PCR Purification Kit (FAVORGEN, Ping Tung, Taiwan). Bidirectional sequencing was carried out using the corresponding forward and reverse primers for *COI* amplification by Macrogen Inc., South Korea.

### Detection of *Leishmania* and other trypanosomatid parasites in the *Culicoides* biting midges

Conventional PCR was employed using the internal transcribed spacer 1 (*ITS1*) region of ribosomal RNA (rRNA) [41]. To detect other trypanosomatids, a nested PCR was performed based on the small subunit (*SSU*) rRNA region [42]. The thermal cycles of the PCR reaction followed the protocol previously described for *Leishmania* spp. [41] and other trypanosomatid parasites [18]. All detection experiments were carried out with PCR components as described in Additional file 1: Supplementary materials 1. Positive PCR products were inserted into the pGEM^®^-T Easy Vector System (Promega, WI, USA), and plasmid extraction was performed and sent for sequencing by Macrogen, Inc., in Seoul, South Korea.

### Identification of blood meal source by host-specific multiplex PCR

Amplification of the mitochondrial cytochrome b (*cytb*) gene was used to detect the presence of vertebrate host-specific DNA in the engorged midges. Multiplex PCR was performed with primers specific to human, cattle, dog, pig, chicken, and universal primers of mammal (UNREV1025) and chicken (UNFOR1029) [43, 44]. Alternative PCR using UNFOR403 and UNREV1025 primers [43, 44] was performed to detect the presence of mammalian DNA in specimens that did not amplify by multiplex PCR. The PCR conditions for mammal blood screening were identical to the multiplex PCR as described in Additional file 1: Supplementary materials 1. Specimens that did not react in either the multiplex PCR or the mammal blood screening were regarded as negative.

### Phylogenetic construction and species delimitation analysis of *Culicoides* biting midges

The *COI* nucleotide sequences obtained were subjected to editing and analysis using the BioEdit sequence alignment editor (version 7.2.5) [45]. The consensus nucleotide sequences were then compared with sequences available in the National Center for Biotechnology Information (NCBI) database using the Basic Local Alignment Search Tool (BLAST) (https://blast.ncbi.nlm.nih.gov/Blast.cgi) and the Barcode of Life Database (BOLD) (https://www.boldsystems.org/). The construction of the phylogenetic tree was achieved by employing the maximum likelihood (ML) method with 1000 bootstrap tests, utilizing Molecular Evolutionary Genetics Analysis software, version 11 (MEGA11) [46]. The ML tree was then visualized and edited using FigTree version 1.4.4 (https://tree.bio.ed.ac.uk/software/FigTree/). The intraspecific variations were calculated using the Kimura 2-parameter (K2P) model in MEGA11 [46].

For the species delimitation, the *COI* sequences of *Culicoides* species were identified at the molecular operational taxonomic unit (MOTU) level generated by the three algorithms: ASAP [Assemble Species by Automatic Partitioning) [47], PTP [Poisson Tree Processes] [48], and TCS [Templeton, Crandall, and Sing] haplotype network [49]. The genetic diversity and neutrality test of *Leishmania* populations and other trypanosomatids were evaluated using DnaSP version 6 [50], and the haplotype network based on the minimum spanning network was then generated using PopART version 1.7 [51].

### Statistical analysis

Data analysis was performed using descriptive statistics based on the PCR-positive results in Microsoft Excel 2019 (Microsoft Corp., USA). The prevalence rate was calculated by dividing the number of positive specimens by the total number of specimens collected in each sampling site, and a 95% confidence interval (95% CI) was evaluated. Other parameters were calculated for the *Culicoides* population from different collection sites, including relative abundance (number of specimens of each species/total number of specimens × 100) and species richness (number of species in the investigated areas).

## Results

### Species composition of *Culicoides* biting midges

**Fig. 2:**
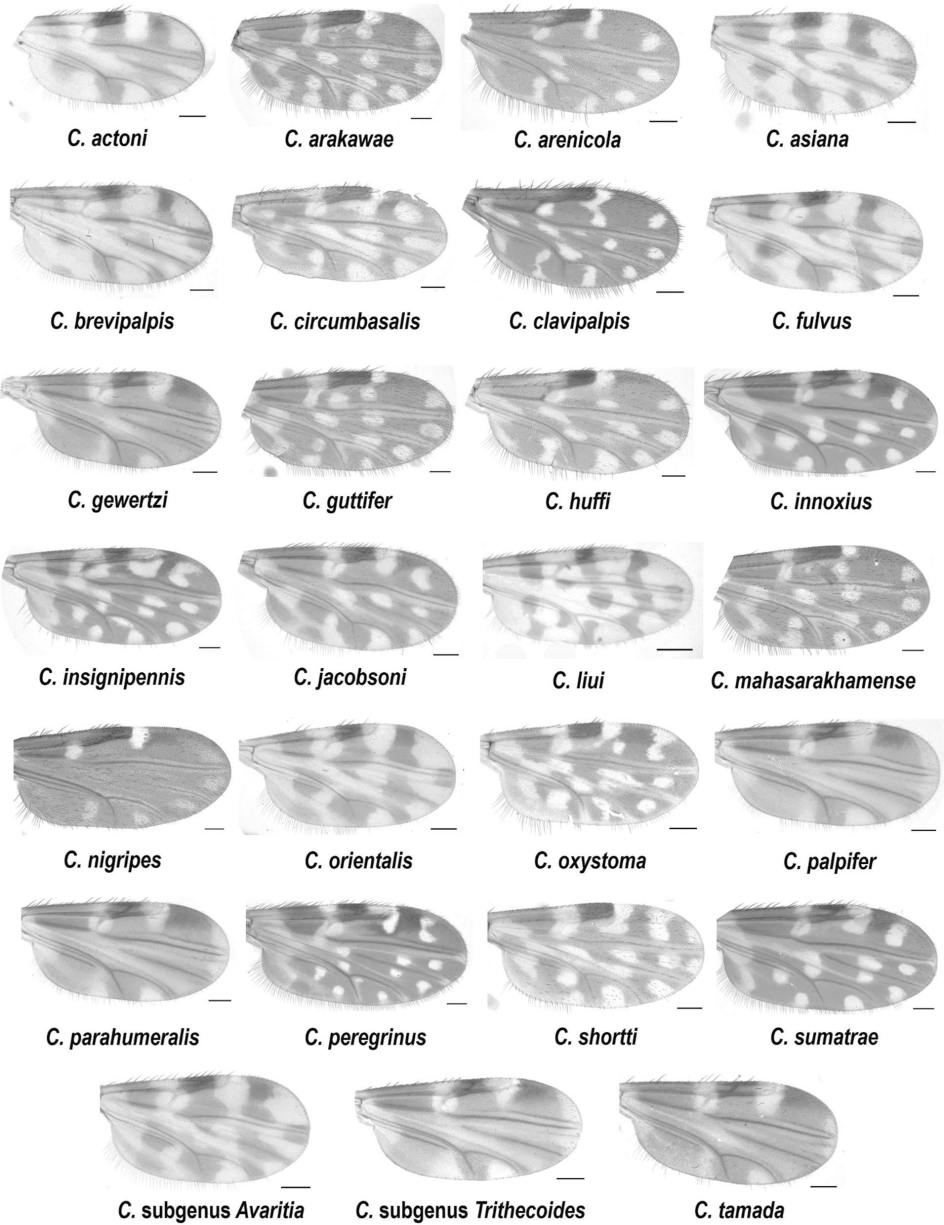
Photographs of wing patterns of representative 27 *Culicoides* species collected from this study; the scale bar indicates 100 µm

Overall, 875 female *Culicoides* biting midges were collected from four different areas in southern Thailand. In total, 716 non-engorged (parous and gravid) female specimens were collected from all sampling sites, whereas 159 blood-fed female midges were collected only in Ron Phibun (NST1). The highest relative abundance of *Culicoides* species in this study was demonstrated in Ron Phibun (56.34%) from Nakhon Si Thammarat, followed by Khlong Thom (24.80%) from Krabi, Phunphin (11.09%) from Surat Thani, and Sichon (7.77%) from Nakhon Si Thammarat. Additionally, the species richness of all collected *Culicoides* spp. was 25, belonging to five subgenera (*Avaritia*, *Hoffmania*, *Meijerehelea*, *Remmia*, and *Trithecoides*) and four species groups (*Clavipalpis*, *Ornatus*, *Shermani*, and *Shortti*). The highest species richness was observed in Ron Phibun (16), followed by Sichon (11), Khlong Thom (11), and Phunphin (10). The most abundant species demonstrated in each study site were *C. peregrinus* (30.03%), *C.* subgenus *Trithecoides* (27.94%), *C. huffi* (72.16%), and *C. guttifer* (29.49%) in Ron Phibun, Sichon, Phunphin, and Khlong Thom, respectively. For the *Trithecoides* subgenus, the specimens were not classified to species level. However, this subgenus was identified by its morphological characteristics of a yellow scutum and three spermathecae. The morphological characteristic wing patterns are illustrated in Fig. 2.

### DNA barcoding and phylogenetic analysis of *COI* sequences

A total of 118 *Culicoides* DNA specimens were randomly selected for *COI* sequencing to represent each morphologically identified species from different sampling sites. Identification of *Culicoides* species using the BOLD search (https://www.boldsystems.org/) demonstrated a success rate for accurate identification. Of the 118 *COI* sequences analyzed, 97 (82.20%) sequences were correctly identified at the species level, while 14 sequences were identified with ambiguity, and seven sequences did not match sequences in the BOLD database. Fourteen ambiguous sequences were identified by morphology into three species, including *C. jacobsoni* (*n* = 7), *C. palpifer* (*n* = 2), and *C.* subgenus *Trithecoides* (*n* = 5). Two ambiguous species (*C. jacobsoni* and *C. palpifer*) were identified at the genus level (*Culicoides* sp.), and five specimens of *C.* subgenus *Trithecoides* were found to be closely related to *Dasyhelea ludingensis* (0.98%) and *Forcipomyia* sp. (0.63%), with relatively low sequence variation. Seven sequences, one from *C. nigripes*, two from *C. palpifer*, and four from *C.* subgenus *Avaritia*, did not match the sequences in BOLD (Additional file 3: Table S1).

A comparison of the two identification methods, BLASTn and BOLD, was conducted in order to confirm the identification of the species. All *COI* sequences were classified to the species level by a BLAST score ranging from 98.25% to 100% similarity, except for *C.* subgenus *Trithecoides* and *C.* subgenus *Avaritia*. BOLD scores ranged from 96.76% to 100% similarity. Three nominal species demonstrated a low percentage identity in BLASTn analysis, with 97.70%, 94.10%, and 91.85% for *C. palpifer*, *C. nigripes*, and *C. clavipalpes*, respectively. In addition, BOLD results demonstrated a low identity percentage for *C. palpifer* (97.26%) and *C. clavipalpes* (95.20%), while *C. nigripes* did not match within the database. Furthermore, BLASTn results for five *C.* subgenus *Trithecoides* specimens showed relatively low percentage identity with Ceratopogonidae species (97.92%), while BOLD results were identical to *D. ludingensis* and *Forcipomyia* sp. *Culicoides* subgenus *Avaritia* showed a low shared identity with *C. fulvus* (88.67%) by BLASTn search; notably, there was no matching of these species in the BOLD database (Additional file 3: Table S1).

**Fig. 3:**
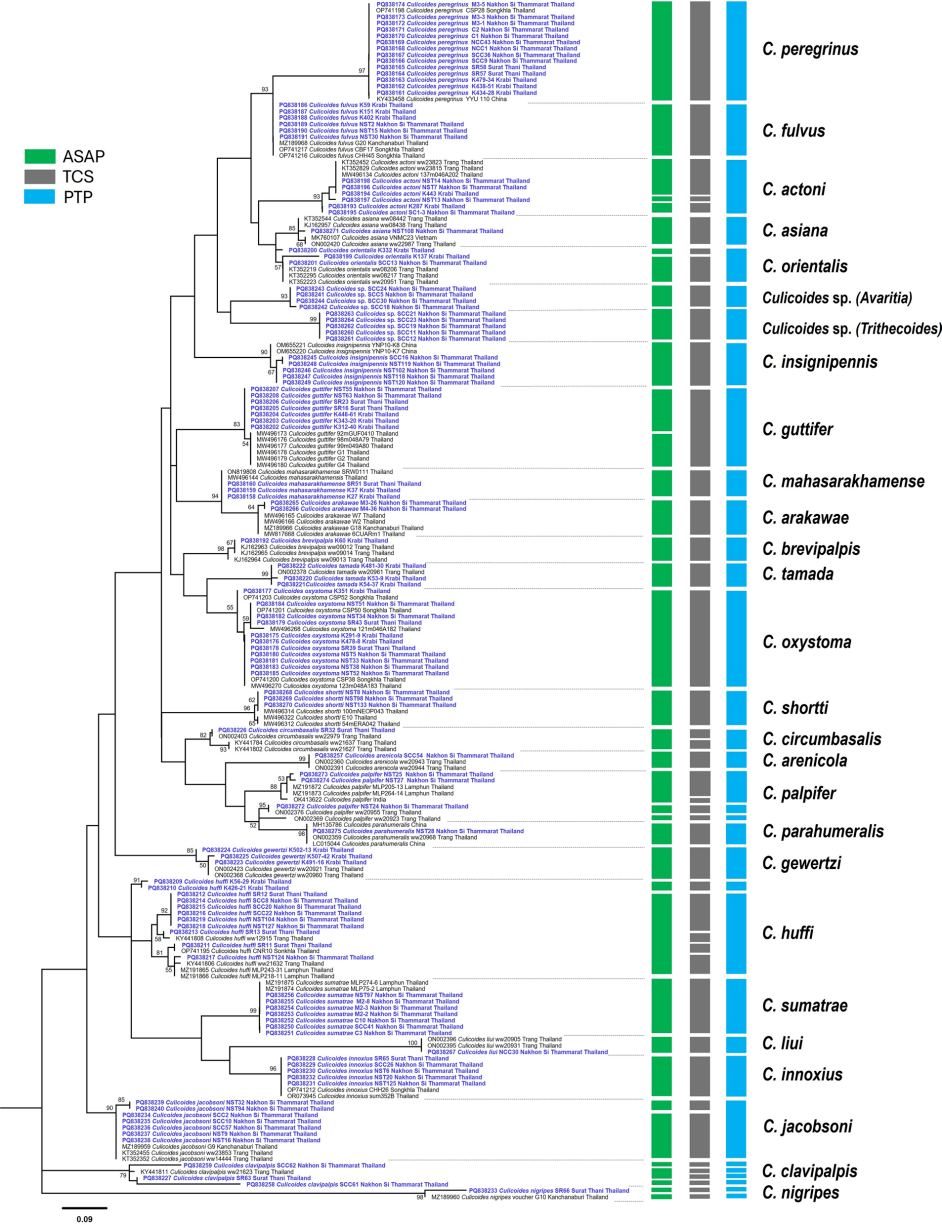
Maximum likelihood tree based on partial *COI* sequences with a Kimura 3-parameter with gamma distribution and invariant sites (T92+G+I) model (1000 bootstrap tests) of 25 *Culicoides* species and their conspecific species and two unidentified *Culicoides* species. The green, gray, and blue bars demonstrate three species delimitation methods—ASAP, TCS, and PTP, respectively

A total of 190 *COI* sequences belonging to 118 of the sequences under investigation and 72 reference sequences of conspecific species in the GenBank database were utilized to construct a phylogenetic tree. Based on the T92+G+I model, the phylogenetic tree has classified our *Culicoides* specimens according to conspecific species with bootstrap tests > 80%. Additionally, the phylogenetic analysis illustrated a distinct separation into two clades of *C. palpifer* and *C. huffi* (Fig. 3). For the K2P intraspecific genetic variations result, the highest maximum intraspecific variation exceeding 3% was demonstrated in five *Culicoides* species, including *C. clavipalpis* (18.80%), *C. huffi* (18.37%), *C. palpifer* (9.64%), *C. orientalis* (8.14%), and *C. actoni* (4.77%) (Additional file 4: Table S2). The *COI* sequences of *Culicoides* species in this study were deposited in the GenBank database as accession numbers PQ838158–PQ838275.

### Species delimitation analysis

Species delimitation was conducted using three methods—ASAP, TCS, and PTP—to demonstrate diversity between species levels by two or more lineages, suggesting a potential cryptic species complex. The results of the three species delimitation methods are shown alongside the ML tree in Fig. 3. The *COI* sequence dataset was sorted into 38, 43, and 34 MOTUs (species groups) by the ASAP, TCS, and PTP methods, respectively. A congruence of ASAP and TCS methods was observed in three species datasets, including *C. actoni* (three MOTUs), *C. orientalis* (two MOTUs), and *C. jacobsoni* (two MOTUs). Furthermore, the ASAP and PTP methods were split into two and three MOTUs in *C. huffi* and *C. palpifer*, whereas the TCS method identified two species, dividing them into four and five MOTUs. The TCS and PTP methods were classified into four MOTUs for *C. clavipalpis*, and the three species delimitation methods were divided into two MOTUs in *C. nigripes*. Furthermore, *C. arakawae* and *C. mahasarakhamense* exhibited analogous wing morphological patterns. However, the three species delimitation methods (ASAP, TCS, and PTP) were clearly distinguishable between the two species. The TCS network analysis of *Culicoides* species in this study demonstrated the presence of 65 haplotypes, with an overall high haplotype diversity (Hd) value of 0.976. The statistical analysis using Tajima's *D* (0.09427) and Fu and Li's *D* (1.14406) was not significant (*P* > 0.10). The highest observed haplotype diversity was found in *C. huffi*, which contained nine distinct haplotypes (H12, H13, H19, H20, H21, H44, H52, H53, and H54). This was followed by *C. palpifer*, which contained six distinct haplotypes (H45, H46, H47, H51, H60, and H51) (Additional file 2: Figure S1.).

### Detection of *Leishmania* spp. and other trypanosomatids in *Culicoides* biting midges

A total of 716 non-engorged biting midges were subjected to molecular screening for *Leishmania* and other trypanosomatid parasites. Overall, 46 (6.42%) of the *Culicoides* biting midges were found to be positive for *Leishmania* DNA. The presence of *Leishmania* DNA was detected at three different sampling sites: Nakhon Si Thammarat (Ron Phibun [NST1] and Sichon [NST2]) and Surat Thani (Phunphin [ST]). Conversely, *Culicoides* biting midges collected from Krabi (KB) were found to be negative for *Leishmania* DNA.

The overall prevalence rate in Ron Phibun (NST1) was 9.28%. *Leishmania orientalis* was detected in *C. peregrinus* (*n* = 18), *C. oxystoma* (*n* = 1), and *C. innoxius* (*n* = 1) with a prevalence rate of 5.98%. *Leishmania martiniquensis* was also detected in *C. peregrinus* (*n* = 9) and *C. oxystoma* (*n* = 2), with a prevalence of 3.29%.

In Sichon (NST2), six specimens of *C. guttifer* (*n* = 2), *C. huffi* (*n* = 2), *C*. subgenus *Avaritia* (*n* = 1), and *C. orientalis* (*n* = 1) were found to be positive for *L. orientalis*, with a prevalence rate of 8.82%.

In Phunphin (ST), six specimens of *C. huffi* were positive for *L. martiniquensis* (6.18%), and one specimen each of *C. guttifer*, *C. innoxious*, and *C. nigripes* was found to contain *L. orientalis* (3.09%), giving an overall prevalence rate of 9.27%.

The BLASTn results of positive specimens for *L. martiniquensis* ranged from 97% to 100% similarity with *L. martiniquensis* reported in humans (accession no. KY982640) from Trang and *Sergentomyia khawi* (accession no. MK603826) from Songkhla provinces of southern Thailand. Conversely, the BLASTn results of positive specimens for *L. orientalis* demonstrated an identity score ranging from 95% to 100% similarity with *L. orientalis* (accession no. MH807723 and MH807729) reported in humans from Trang province of southern Thailand.

The present study detected monoxenous trypanosomatids with the primer targeted to the *SSU* rRNA gene, with an overall prevalence rate of 1.82% (13/716). Eleven *C. peregrinus* specimens (3.29%) were positively detected in Ron Phibun (Nakhon Si Thammarat). Sequence comparison of the 11 positive specimens with data available in GenBank revealed high sequence similarity (99–100%) with *SSU* rRNA sequences of *Crithidia* sp. (accession no. OR077444, OR077445, and OR077454) found in *C. peregrinus* as previously reported in Thailand. Furthermore, two (0.92%) specimens of *C.* subgenus *Trithecoides* collected from Krabi province were positively detected. A comparison of the two obtained *SSU* rRNA sequences with sequences published in the GenBank database demonstrated that the sequences were 98.92% and 98.93% compatible with *Crithidia brevicula* (accession no. MT232052). However, co-infection of *Leishmania* spp. and *Crithidia* spp. could not be detected (Additional file 5: Table S3).

The obtained sequences were submitted to the GenBank database under accession numbers PQ805171–PQ805187 for *L. martiniquensis*, PQ805188–PQ805216 for *L. orientalis*, PQ799320–PQ799330 for *Crithidia* sp., and OP315268 and OP315269 for *.Crithidia brevicula* (Table 1).

**Table 1:** Species composition, number of specimens, relative abundance, and species richness in different locations in southern Thailand

Subgenus/species group	*Culicoides* species	Sampling sites					Total
		NST1		NST2	ST	KB	
		Unfed (*n*)	Blood-fed (*n*)	Unfed (*n*)	Unfed (*n*)	Unfed (*n*)	
*Avaritia*	*C. actoni*	8	2	0	0	4	14
*Avaritia*	*C. asiana*	1	0	0	0	0	1
*Avaritia*	*C. brevipalpis*	0	0	0	0	3	3
*Avaritia*	*C. fulvus*	4	3	0	0	7	14
*Avaritia*	*C. jacobsoni*	12	14	4	0	0	30
*Avaritia*	*C. orientalis*	0	12	4	0	15	31
*Avaritia*	*Culicoides* sp.	0	0	5	0	0	5
* Clavipalpis* group	*C. arenicola*	0	0	1	0	0	1
* Clavipalpis* group	*C. clavipalpis*	0	0	4	1	0	5
* Clavipalpis* group	*C. huffi*	13	1	11	70	48	143
*Hoffmania*	*C. innoxius*	20	25	3	1	0	49
*Hoffmania*	*C. insignipennis*	19	18	3	0	0	40
*Hoffmania*	*C. liui*	1	0	0	0	0	1
*Hoffmania*	*C. peregrinus*	138	10	2	3	61	214
*Hoffmania*	*C. sumatrae*	12	12	1	0	0	25
*Meijerehelea*	*C. arakawae*	2	0	0	0	0	2
*Meijerehelea*	*C. guttifer*	11	0	10	5	64	90
*Meijerehelea*	*C. mahasarakhamense*	0	0	0	1	2	3
*Ornatus* group	*C. cricumbasalis*	0	0	0	1	0	1
*Remmia*	*C. oxystoma*	79	5	1	13	5	103
*Shermani* group	*C. nigripes*	0	0	0	1	0	1
*Shortti* group	*C. shortti*	3	1	0	0	0	4
*Trithecoides*	*C. gewertizi*	0	0	0	0	3	3
*Trithecoides*	*C. palpifer*	3	0	0	0	0	3
*Trithecoides*	*C. parahumeralis*	1	0	0	0	0	1
*Trithecoides*	*C. tamada*	0	0	0	0	3	3
*Trithecoides*	*Culicoides* sp.	7	56	19	1	2	86
Subtotal		334	159				
Total		493		68	97	217	875
Relative abundance		56.34		7.77	11.09	24.80	
Species richness^a^		16		11	10	11	25

NST1: Nakhon Si Thammarat, Ron Phibun district, NST2: Nakhon Si Thammarat, Sichon district, ST: Surat Thani, KB: Krabi

^a^All identified species were used for calculation, except for *Culicoides* sp. (subgenera *Avaritia* and *Trithecoides*)

### Haplotype network and genetic diversity of *Leishmania *and *Crithidia* parasites

**Fig. 4:**
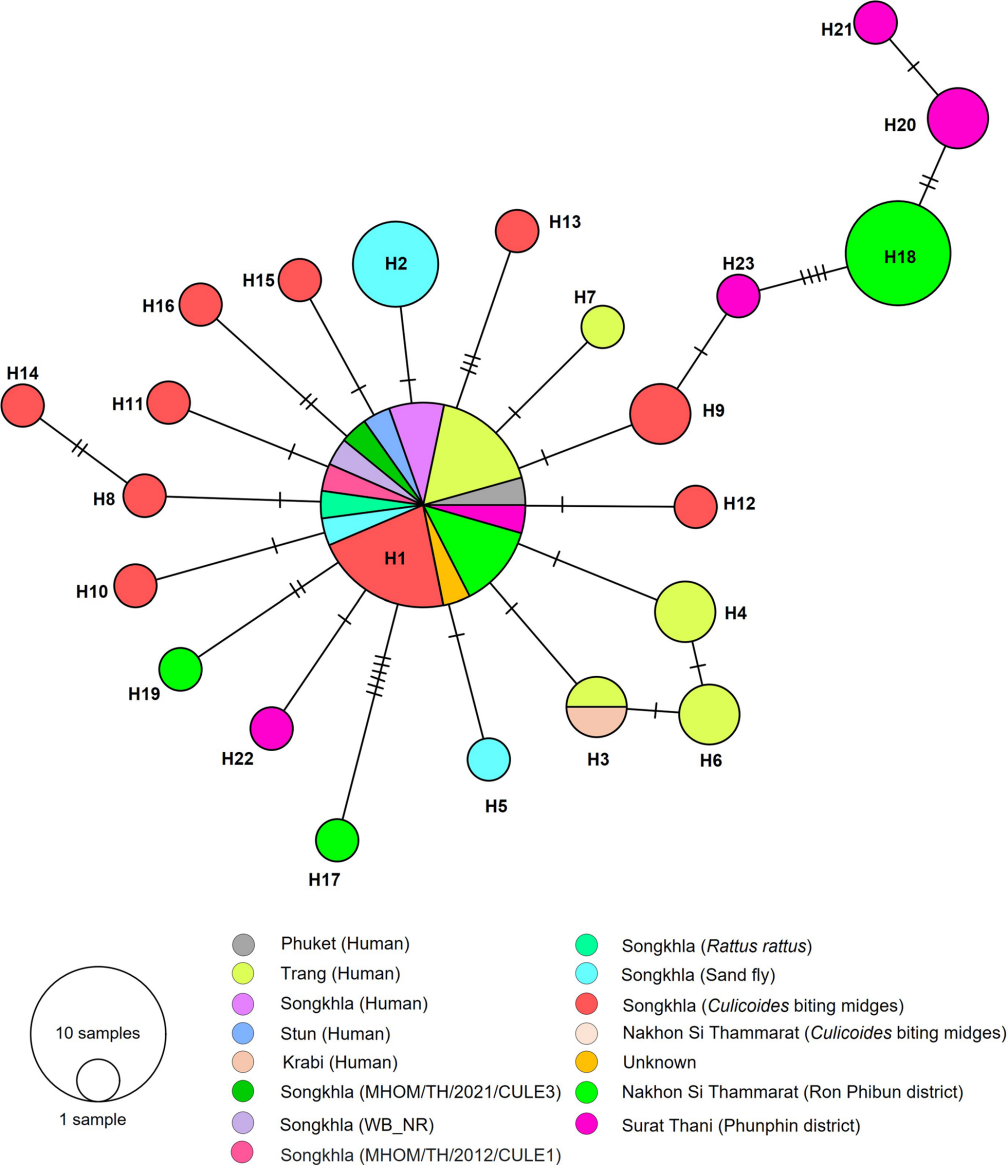
Haplotype diversity network of *L. martiniquensis* based on *ITS1* sequences from our collected *Culicoides* biting midges and *L. martiniquensis* sequences from different hosts from southern Thailand. The size of each circle is proportional to the frequency of the respective haplotype. The lines in the branch correspond to the number of mutations

The present study investigates the genetic diversity of *L. martiniquensis*, utilizing a dataset comprising 58 *ITS1* sequences derived from *Culicoides*-positive specimens and those previously reported in southern Thailand from human, sand fly, rat, and biting midge specimens. Twenty-three distinct haplotypes were identified, encompassing 33 polymorphic sites. The classification of our *Leishmania* sequences revealed their association with eight distinct haplotypes, namely H1, H17, H18, and H19 from Nakhon Si Thammarat (NST1), and H20, H21, H22, and H23 from Surat Thani. H1 was identified as the predominant haplotype, comprising 23 sequences from multiple hosts (Fig. 4). The haplotype diversity (Hd) was relatively high, at 0.831. In contrast, the nucleotide diversity (π) was relatively low at 0.01224. The neutrality test was found to be negatively significant for Tajima's *D* (−1.89378) and Fu and Li's *D* (−3.95199) (Table 2).

**Table 2:** Genetic diversity of *Leishmania* and *Crithidia* parasites from *Culicoides* species

Parasites	No. of sequences (*N*)	Genetic diversity					Neutrality tests	
		No. of haplotype (H)	No. of polymorphic sites (S)	Average of number of nucleotide differences (*k*)	Haplotype diversity (Hd ± SD)	Nucleotide diversity (π ± SD)	Tajima’s *D*	Fu and Li’s *D*
*L. martiniquensis*	58	23	33	3.12220	0.831 ± 0.046	0.01224 ± 0.00181	−1.89378*	−3.95199**
*L. orientalis*	63	25	56	4.54583	0.726 ± 0.063	0.01951 ± 0.00480	−2.15392*	−2.81159*
*Crithidia* spp.	67	31	75	19.78200	0.941 ± 0.016	0.03590 ± 0.00164	0.51741***	−0.07122***

*P < 0.05, **P < 0.02, ***Not significant, *SD* standard deviation

**Fig. 5:**
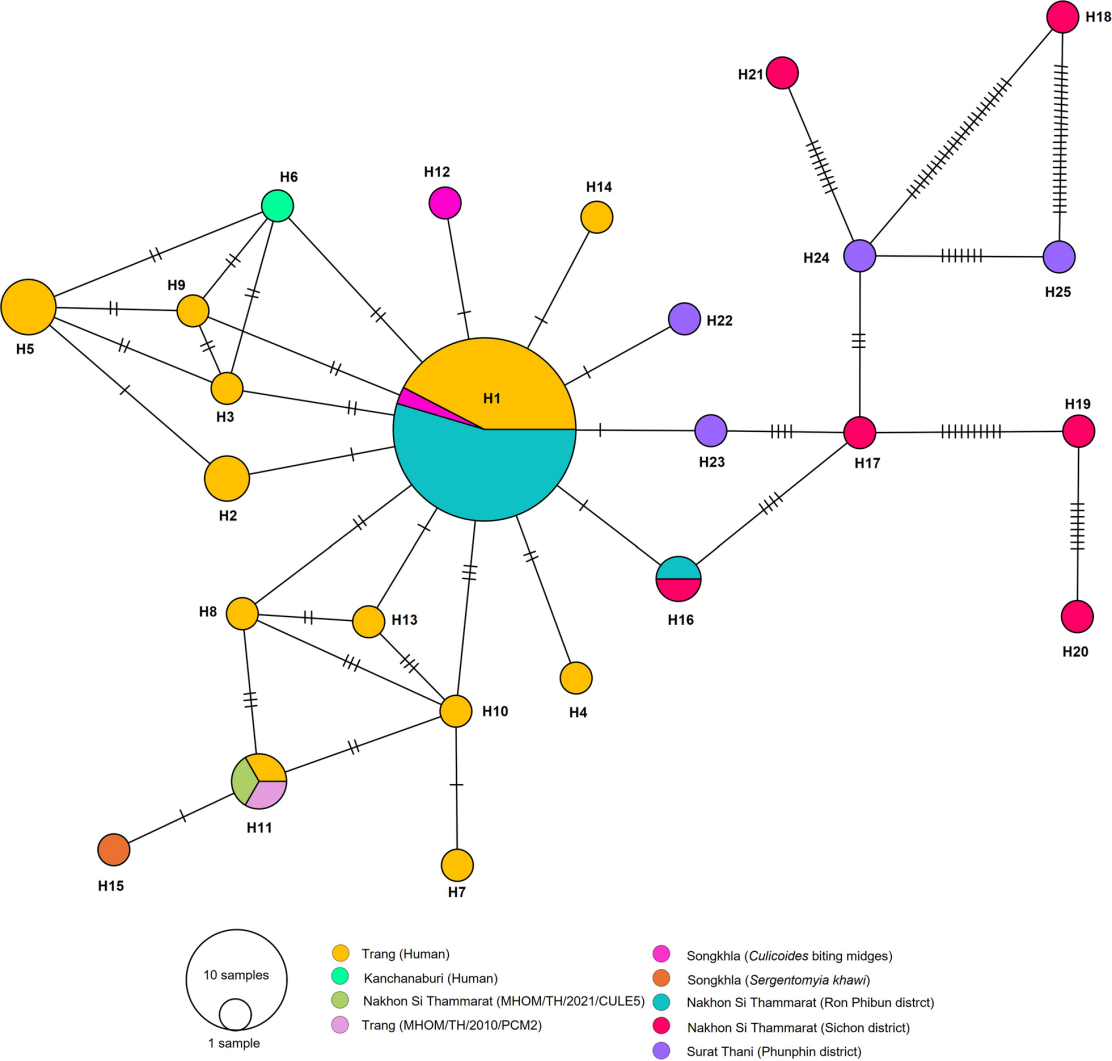
Haplotype diversity network for *L. orientalis ITS1* sequences detected in *Culicoides* biting midges, compared with *L. orientalis* as previously reported from southern Thailand. The size of the circles is proportional to the number of specimens. Each line in the branches indicates the number of base mutations

A total of 63 *L. orientalis ITS1* sequences, including our *Culicoides* specimens, along with those sequences from humans, sand flies, and biting midges previously reported in southern Thailand, were utilized for genetic and haplotype analysis. Twenty-five distinct haplotypes were identified, encompassing 56 polymorphic sites. Our *L. orientalis* was identified within 11 haplotypes, consisting of six haplotypes (H16, H17, H18, H19, H20, and H21) from Sichon (NST2), four haplotypes (H22, H23, H24, and H25) from Surat Thani, and two haplotypes (H1 and H16) from Ron Phibun (NST1). The predominant haplotype of *L. orientalis* was H1, comprising 33 sequences from human (*n* = 14), *Culicoides* biting midges from Songkhla (*n* = 1), and our sequences from Ron Phibun (NST1) (*n* = 18) (Fig. 5). The genetic diversity of *L. orientalis* exhibited a high haplotype diversity (Hd = 0.726) and a relatively low nucleotide diversity (*π* = 0.01951). The neutrality test yielded negative results, with Tajima's *D* and Fu and Li's *D* values of −2.15392 and −2.81159, respectively (Table 2).

**Fig. 6:**
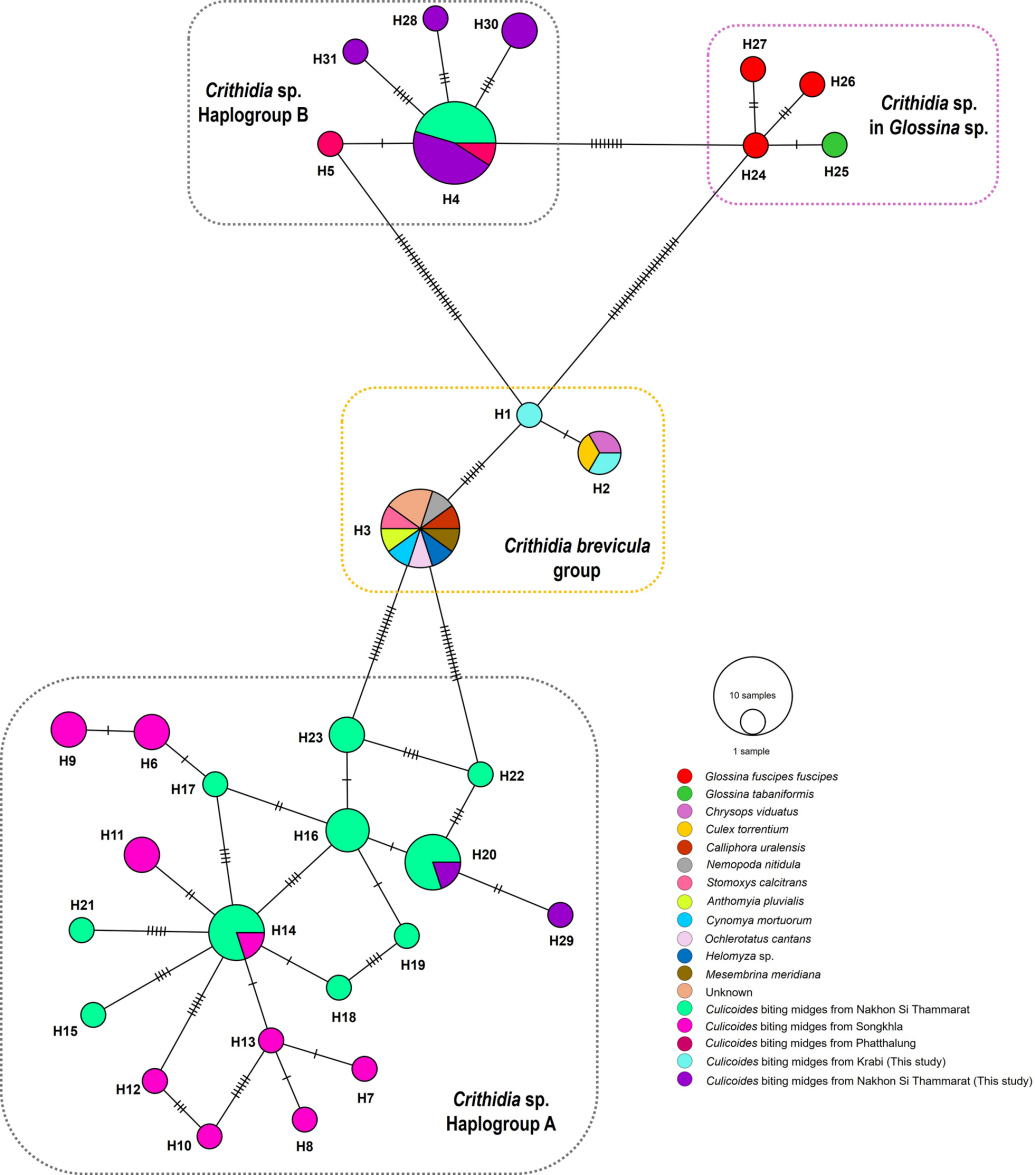
The haplotype diversity for *Crithidia SSU* rRNA sequences detected in *Culicoides* biting midges of this study and sequences of different insect species is demonstrated in this following analysis. The size of each circle is proportional to the number of specimens. The line in the branches demonstrates the number of base mutations

For *Crithidia* sp., 67 *SSU* rRNA sequences were used for haplotype and genetic analysis. Of these, 31 classified haplotypes, which were divided into four distinct groups: * Crithidia brevicula* group, *Crithidia* sp. in *Glossina* sp. group, *Crithidia* haplogroup A, and *Crithidia* haplogroup B. Notably, 11 *SSU* rRNA sequences isolated from this study were classified into two major haplogroups, A and B. For *Crithidia* haplogroup A, there are 19 haplotypes belonging to *Crithidia* sequences previously reported in *Culicoides* biting midges from Songkhla and Nakhon Si Thammarat, and our sequences. The H20 and H29 were identified in our *Crithidia* species. The *Crithidia* haplogroup B was classified into five distinct haplotypes, demonstrating one (H4) belonging to *Crithidia* sp. from Nakhon Si Thammarat and two (H4 and H5) from Phatthalung provinces, while four *Crithidia SSU* rRNA sequences in this study were classified into four distinct haplotypes, namely H4, H28, H30, and H31. For * Crithidia brevicula*, three distinct haplotypes were identified. *Crithidia brevicula* sequences from Krabi were grouped into H2 by one sequence, sharing with * Crithidia brevicula* from *Culex torrentium* and *Chrysops viduatus*, while the remaining sequence belonged to H1, which was a unique haplotype (Fig. 6). The genetic diversity of *Crithidia* was found to be 0.941, suggesting high haplotype diversity. In contrast, the nucleotide diversity was 0.03590, indicating a relatively low level. The neutrality tests were not significant in the populations (Table 2; Additional file 6: Table S4).

### Host blood meal identification in *Culicoides* biting midges

A total of 159 blood-fed specimens were collected, representing 11 species (*C. actoni*, *C. fulvus*, *C. huffi*, *C. innoxius*, *C. insignipennis,* C. *jacobsoni*, *C. orientalis*, *C. oxystoma*, *C. peregrinus*, *C. shortti*, and *C. sumatrae*) and one subgenus, *Trithecoides*. These specimens were then tested for the presence of host DNA. Of these, two specimens were found to be negative in both the multiplex PCR and the mammalian blood screening PCR. The multiplex PCR demonstrated an efficacy rate of 98.74% (157/159) in host-specific identification. The most prevalent blood meal pattern identified in this study was cow blood DNA in 149 (94.90%) specimens of 12 *Culicoides* species. In addition, dog blood DNA was identified in three (1.91%) *C*. subgenus *Trithecoides*. Notably, only one specimen (0.64%) of *C. huffi* was positive for chicken blood DNA. Interestingly, four specimens were found to be mixed with host blood DNA; one specimen of *C. innoxius* was fed on both cows and chickens. Furthermore, each specimen of *C. orientalis*, *C. oxystoma*, and *C. sumatrae* was found to be feeding on both humans and cows. Conversely, no specimen was found to be positive for pig blood DNA (Additional file 7: Table S5).

## Discussion

Morphological identification of *Culicoides* species has historically relied on keys and taxonomic descriptions. However, this method has several limitations, including the requirement for expertise, intraspecific morphologic variation, and the fact that specimens are often damaged during collection or preservation [37, 52], leading to misidentification. DNA barcoding has been increasingly applied for *Culicoides* identification, providing high precision in species identification, especially for cryptic species that are morphologically indistinguishable [30, 52]. In Thailand, 103 species of *Culicoides* biting midges have been recorded [25, 32, 53, 54]. Several investigations have been conducted to identify *Culicoides* species in different areas of Thailand [21, 22, 30–32]. This study investigated the diversity of *Culicoides* biting midges based on morphological characteristics and DNA barcoding for species delimitation in leishmaniasis areas of southern Thailand. Twenty-five *Culicoides* species were identified based on morphological traits and confirmed at the species level by *COI* sequencing. Furthermore, two subgenera were identified at the genus level, *Trithecoides* and *Avaritia.* The predominant *Culicoides* species at the four sampling sites were *C. peregrinus*, *C*. subgenus *Trithecoides*, *C. huffi*, and *C. guttifer*. These were found in Ron Phibun, Sichon, Phunphin, and Khlong Thom, respectively. Higher species richness (SR) was found in Ron Phibun (SR = 16), followed by Sichon and Khlong Thom (SR = 11) and Phunphin (SR = 10). Previous studies of *Culicoides* species composition in leishmaniasis endemic areas of southern Thailand demonstrated that *C. peregrinus* was the most common species, with 17 species found in Phatthalung province [24], while *C. peregrinus* and *C. oxystoma* were the dominant species with seven and six species in the two areas in the Sadao district, and *C. fordae* was the dominant species with 10 species found in the Ratthaphum district in Songkhla province [22]. A study by Kaewmee et al. demonstrated low species richness (*C. peregrinus*, *C. mahasarakhamense*, and *C. oxystoma*) in Nakhon Si Thammarat [23]. The variations in dominant species and species richness across the designated areas may be attributable to several factors, including seasonality, environmental factors (temperature, rainfall, wind speed, and humidity), host animal availability, and the optimal height and distance of traps with animal baits [55, 56].

The *COI* sequences of *Culicoides* species in this study were verified in the BOLD database, indicating an 82.20% (97/118) success rate for correct identification. However, 14 sequences were identified as ambiguous, and seven sequences had no match in the BOLD database. The failure to identify the species may be attributed to misidentification of the specimens in public databases or to sequence variations of the *COI* gene, which cannot distinguish some conspecific species [30]. Phylogenetic analysis clearly delineates two and three lineages of *C. palpifer* and *C. huffi*, respectively. These data are consistent with the results of previous studies on potential cryptic species of *C. palpifer* and *C. huffi* collected from the leishmaniasis area in Lamphun, northern Thailand  [21], and the specimens from southern and eastern Thailand [34].

In a recent study, Jomkumsing et al. reported the possible existence of cryptic species for five species: *C. arakawae*, *C. huffi*, *C. innoxius*, *C. jacobsoni*, and *C. actoni*. These findings were derived from a combination of phylogenetic neighbor-joining (NJ) tree analysis and ABGD (Automatic Barcode Gap Discovery) analysis, a method that has proven effective in identifying genetic lineage variations. [30]. This study employed species delimitation methods, including ASAP, TCS, and PTP, to demonstrate the presence of cryptic species of *Culicoides* biting midges based on single-locus data from *COI* sequences. Six species, *C. actoni*, *C. orientalis*, *C. huffi*, *C. palpifer*, *C*. *clavipalpis*, and *C. jacobsoni*, were identified as exhibiting two or more MOTUs. Similarly, intraspecific genetic variation analysis showed high divergence in five species, namely, *C. clavipalpis* (18.80%), *C. huffi* (18.37%), *C. palpifer* (9.64%), *C. orientalis* (8.14%), and *C. actoni* (4.77%). This finding suggests the potential existence of a cryptic species complex. Additionally, all species delimitation methods and phylogenetic analysis supported the proposal of a cryptic species complex within *C. palpifer* and *C. huffi*, as reported in previous studies [21, 30, 34]. Furthermore, other species, *C. actoni*, *C. clavipalpis*, and *C. jacobsoni*, were consistent with the findings of previous studies by Fujikawa et al. [31]. In the case of *C. nigripes*, this species demonstrated low percentage identity (94.10%) in the BLASTn result and no match sequence within the BOLD database. In contrast, three species delimitations, ASAP, TCS, and PTP, were separated by two specimens. However, the single sequence of *C. nigripes* deposited in the GenBank database (MZ189960) was insufficient for comparative analysis, as it was only reported from Kanchanaburi, western Thailand. It is, therefore, imperative to collect additional specimens from different locations for this species in order to facilitate further analysis. *Culicoides arakawae* and *C. mahasarakhamense* exhibited similar wing morphological patterns [30, 53], which made it difficult to distinguish between the two species based solely on their external features. However, when employing the three species delimitation methods—ASAP, TCS, and PTP—a clear genetic separation between the two species was observed. This finding underscores the limitations of relying solely on morphological traits for species identification, particularly in cases of cryptic or closely related species.

In the present study, the identification of *C.* subgenus *Trithecoides* and *C.* subgenus *Avaritia* specimens collected from Sichon, Nakhon Si Thammarat, was conducted based on fundamental morphological characteristics, utilizing BLASTn, BOLD, phylogenetic analysis, and species delimitation. The inability to assign these specimens to the species level led to the hypothesis that they might not possess any sequences from the two subgenera (*Trithecoides* and *Avaritia*) available in the database, or they could be a novel species. However, further investigation into the taxonomic identification of these insects, based on whole bodies, may be conducted in the future. In this study, morphological and *COI* barcoding were conducted, demonstrating that DNA barcoding can effectively confirm species and identify potential cryptic species. It can also be used to confirm species in cases where there is a lack of expertise or when insect specimens are incomplete.

Molecular and morphological methods for identifying *Culicoides* species involved in leishmaniasis are highly complementary, with each addressing the limitations of the other. Morphological identification using traits such as wing venation, genitalia, and body structure is often used in field settings where only basic tools are available, but it can be difficult to distinguish closely related or cryptic species. Molecular techniques, such as DNA barcoding using the *COI* gene, provide accurate species identification and can confirm or clarify ambiguous morphological findings, especially when cryptic species are involved [30, 34]. In situations where only morphological methods are feasible, molecular identification can still play a vital role when specimens are returned to the laboratory, ensuring accuracy and revealing species that may have been misidentified in the field. Integrating molecular data with morphological identification not only improves the overall accuracy of species identification but also enhances surveillance efforts, allowing for better tracking of *Culicoides* populations involved in leishmaniasis transmission and informing more targeted control measures.

In the present study, natural infections by *Leishmania* parasites were conducted in four sampling sites from three provinces, including Nakhon Si Thammarat, Krabi, and Surat Thani in southern Thailand. All selected provinces have reported several cases of autochthonous leishmaniasis caused by *Mundinia* species. The molecular evidence of *Leishmania* infection in potential vectors was performed by *ITS1*-PCR-based detection. The study revealed the sympatric infections of *L. martiniquensis* and *L. orientalis* in *C. peregrinus* and *C. oxystoma*, with the detection of *L. orientalis* in a single specimen of *C. innoxius* from Ron Phibun. Our result was consistent with the previous study by Songumpai et al., which mentioned the co-circulation of *L. martiniquensis* and *L. orientalis* in *C. peregrinus* and *C. oxystoma* from Songkhla in southern Thailand. [22].

In the present study, *C. peregrinus* was identified as the predominant species and was found to be positive for *Leishmania* detection in Ron Phibun. A previous study by Kaewmee et al. suggested that *C. peregrinus* is a potential vector of *L. martiniquensis* from southern Thailand [23]. *Leishmania orientalis* was detected in *C. guttifer*, *C. huffi*, *C. orientalis*, and *C.* subgenus *Avaritia* in Sichon, while *L. martiniquensis* was not detected. The high prevalence of *Leishmania* infection was predominantly observed in *C. peregrinus* (NST1) and *C. huffi* (in NST2 and ST). It was hypothesized that the most abundant *Culicoides* species are potential vectors for increasing disease transmission of pathogens [22, 57]. Previous studies have reported that several *Culicoides* species were detected for *L. martiniquensis* DNA, including *C. mahasarakhamense*, *C. peregrinus*, *C. oxystoma* [22, 23], *C*. *huffi*, *C. fordae*, and *C. fulvus* [22], in southern Thailand. Consistent with the present study, *L. martiniquensis* was positive in *C. peregrinus*, *C. oxystoma*, and *C. huffi*. While *C. peregrinus* and *C. oxystoma* have been documented as positive for *L. orientalis* [22], our study presents the novel finding of molecular evidence for *L. orientalis* in *C. guttifer*, *C. huffi*, *C. orientalis*, *C*. *innoxius*, and *C. nigripes* in the southern region of Thailand. However, recent studies by Soe et al. have shown a remarkable inability to detect *Leishmania* and other trypanosomatid DNA in *Culicoides* biting midges sampled from mixed livestock farms in Nakhon Si Thammarat [58]. These findings raise questions about the ecological and environmental factors influencing the presence or absence of these pathogens in such vectors and suggest a need for further investigation to understand the dynamics of vector–pathogen interactions in different geographic settings. Previous reports indicated that traditional vectors such as sand flies in northern and southern Thailand were found to be positive for *L. orientalis* in *Sergentomyia iyegari* [59], *Se. gemmea* [19], and *Se. khawi* [60]. Additionally, *L. martiniquensis* was reported in *Se. khawi* [18], *Se. gemmea*, *Se. barraudi* [17], *Grassomyia indica* [20], and *Phlebotomus stantoni* [19].

The genetic diversity of both *L. martiniquensis* and *L. orientalis* populations was found to be characterized by high haplotype diversity and relatively low nucleotide diversity, suggesting that they have recently diverged in the populations [35, 36, 61]. A star-like or complex distribution pattern was demonstrated by the haplotype network of both species, with the origin haplotype and several connecting haplotype lineages with a few base mutations, indicating a recent population expansion from the major haplotype [62]. Furthermore, a significant negative result of the neutrality test suggested possible rapid population growth or selective sweep [36, 61, 63]. Of particular significance is the observation that the genetic divergence of novel haplotypes may be indicative of an evolutionary process by which parasites have successfully adapted to a diverse range of vector species or may be associated with a separate vector. [62, 63].

The detection of blood meal provides information about the host preferences of potential leishmaniasis vectors. In the present study, 159 biting midges from Ron Phibun in Nakhon Si Thammarat were blood-engorged. Our findings revealed that the primary blood source for these biting midges was cows, constituting 94.90% of the samples, followed by dogs (1.91%). This observation indicates a strong mammalophilic blood preference and underscores the potential role of these mammal species as reservoirs for disease transmission. Furthermore, mixed blood-feeding of humans and cows was found in *C. orientalis*, *C. oxystoma*, and *C. sumatrae*, while *C. innoxius* was found to feed on both cows and chickens. In Thailand, previous reports on blood meal analysis in *Culicoides* were mainly conducted in different locations [24, 30, 32, 64]. The findings revealed that the host preferences of midges depend primarily on the availability of animals [30, 32]. A few studies in Thailand have identified an anthropophilic blood preference in *C. brevitarsis* [32], *C. oxystoma*, and *C. imicola* [64]. However, in the present study, three species, *C. orientalis*, *C. oxystoma*, and *C. sumatrae*, were shown to feed on human blood. Of particular interest is the positive detection of *C. oxystoma* for both *L. martiniquensis* and *L. orientalis* in the present study, as well as in a previous study conducted in southern Thailand [22]. Consequently, it is hypothesized that *C. oxystoma* may act as a potential vector for transmission to humans or animals.

The detection of parasite DNA and the identification of human blood in field-caught biting midges supported only two of Killick-Kendrick's criteria [65]. It is impossible to incriminate *Culicoides* as the principal vector of leishmaniasis in Thailand. The remaining criteria include the isolation and cultivation of these parasites from insect vectors in the field, the experimental infection of parasites for completed development cycles in Thai *Culicoides* species, and the transmission of parasites to vertebrate hosts by bite. However, the missing evidence data will be provided for completion in future studies to fulfill the criteria.

Furthermore, the utilization of *SSU* rRNA-PCR for the screening of trypanosomatids in biting midges resulted in the identification of *Crithidia* sp. in *C. peregrinus* from Ron Phibun, Nakhon Si Thammarat province, and the detection of *Crithidia brevicula* in two *C*. subgenus *Trithecoides* specimens in Khlong Thom, Krabi province. These parasites are one-host or monoxenous trypanosomatids and are commonly found in the intestinal tract of insects [66]. A previous study by Sunantaraporn et al. reported *Crithidia* sp. in *C. peregrinus* from Phatthalung province in southern Thailand [24]. Furthermore, *Crithidia* sp. was identified in several *Culicoides* species, including *C. peregrinus*, *C. orientalis*, *C. oxystoma*, *C. fordae*, and *C. elbeli*, collected from Songkhla in southern Thailand [22]. A subsequent study by Kaewmee et al. identified two distinct clades (designated as A and B) of *Crithidia* spp. in *C. peregrinus* specimens obtained from Nakhon Si Thammarat in southern Thailand [23]. Previous research in eastern Lithuania had identified *Crithidia* sp. in *C. festivipennis*, and * Crithidia brevicula* had been found in *C. festivipennis*, *C. kibunensis*, and *C. pictipennis* [67]. In the present study, we report *Crithidia brevicula* in *C*. subgenus *Trithecoides* in Thailand for the first time. In contrast, *Crithidia brevicula* has been observed to infect a broader range of species, including insects from the families Nabidae, Diptera, and Muscidae [68]. The analysis of *Crithidia* sp. detection was classified into two haplogroups, A and B. Of particular interest was the finding that haplogroup A contained *Crithidia* sp. from various *Culicoides* species, including *C. peregrinus*, *C. orientalis*, *C. oxystoma*, *C. fordae*, and *C. elbeli*, while haplogroup B contained *Crithidia* sp. from only one species of *C. peregrinus*, suggesting that *Crithidia* haplogroup B may be specific to *C. peregrinus*. However, the isolation of these trypanosomatids was not performed in this study. Therefore, it is recommended that future research focus on observing live parasites in biting midges to gain a deeper understanding of their biology and interactions.

## Conclusions

The findings of this study provide compelling evidence of the presence of mixed-blood hosts and the co-circulation of *L. martiniquensis* and *L. orientalis* within *Culicoides* populations in leishmaniasis-endemic areas of southern Thailand. Additionally, the study identified cryptic species of *Culicoides* biting midges, highlighting the complexity of vector species in the region. The results support the hypothesis that *Culicoides* may act as vectors for *Leishmania* transmission, thereby contributing to the zoonotic transmission risk in this area. The diversity of *Culicoides* species further underscores the importance of understanding vector species composition as a key factor influencing disease transmission dynamics. It is recommended that future research focus on investigating the role of *Culicoides* species as vectors of *Leishmania* in southern Thailand. Additionally, longitudinal studies should be conducted to assess seasonal variations in vector populations and their implications for disease transmission. Expanding the study to other regions with similar environmental conditions will provide a broader understanding of the geographic spread and risk factors associated with *Leishmania* transmission in Thailand. Enhanced surveillance and vector control strategies are also recommended to mitigate the risk of leishmaniasis transmission.

## Supplementary Information

Additional file 1: Supplementary materials 1.

Additional file 2: Figure S1. The TCS haplotype network of *COI* sequences of *Culicoides* biting midges from this study.

Additional file 3: Table S1. Morphological identification and nucleotide search identified results in the BOLD and BLAST databases of *Culicoides* species collected from this study.

Additional file 4: Table S2. K2P intraspecific genetic divergences of *Culicoides* species were collected in this study.

Additional file 5: Table S3. *Culicoides* species identification, number of positive specimens for *Leishmania*, and other trypanosomatids collected from four different areas in southern Thailand.

Additional file 6: Table S4. The haplotype distribution data of *ITS1* sequences of *L. martiniquensis* and *L. orientalis* and *SSU* rRNA sequences of *Crithidia* spp.

Additional file 7: Table S5. Host blood meal identification of *Culicoides *biting midges collected from Ron Phibun district in Nakhon Si Thammarat, southern Thailand.

## Data Availability

The data supporting the findings of the study must be available within the article and/or its supplementary materials, or deposited in a publicly available database. Please revise the data availability statement accordingly. The sequence data obtained from this study have been deposited in the GenBank database (https://www.ncbi.nlm.nih.gov/genbank/). All sequences are available in GenBank under the accession numbers PQ805171–PQ805187 for *L. martiniquensis*, PQ805188–PQ805216 for *L. orientalis*, PQ799320–PQ799330 for *Crithidia* sp., and OP315268 and OP315269 for *Crithidia brevicula*. PQ838158–PQ838275 for *Culicoides* species are based on the *COI* gene.
