# Supplementary material for: DNA barcoding of Culicoides biting midges (Diptera: Ceratopogonidae) and detection of Leishmania and other trypanosomatids in southern Thailand
Source: Parasit Vectors. 2025 May 29;18:194. doi: 10.1186/s13071-025-06812-0 (PMC12121006; doi:10.1186/s13071-025-06812-0)
Supplement: Supplementary file 1 — Additional file 1: Supplementary materials 1. [file 13071_2025_6812_MOESM1_ESM.pdf]

Supplementary material 1.

Detection of *Leishmania* and other trypanosomatid parasites in the *Culicoides* biting midges

Conventional PCR was employed using the internal transcribed spacer 1 (*ITS1*) region of ribosomal RNA (rRNA) with forward primer L5.8S inner (5′-GTTATGTGAGCCGTTATCC-3′) and reverse primer LITSR2 (5′-CTGGATCATTTTCCGATGATT-3′), which produced a product of DNA fragment approximately 272-280 bp [1]. To detect other trypanosomatids, a nested PCR was performed based on the small-subunit (*SSU*) rRNA region using outer primers, TRY927F (5′-GAAACAAGAAACACGGGAG-3′) and TRY927R (5′-CTACTGGGCAGCTTGGA-3′). The inner primers utilized were SSU561F (5′-TGGGATAACAAAGGAGCA-3′) and SSU561R (5′-CTGAGACTGTAACCTCAAAGC-3′), resulting in the amplification of a product with an approximate size of 560 bp [2]. The thermal cycles of the PCR reaction followed the protocol previously described for *Leishmania* sp. [1] and other trypanosomatid parasites [3]. All detection experiments were carried out with PCR components, comprising 12.5 μL of 2× Hot-Start PCR Master Mix (Biotechrabbit, Berlin, Germany), 0.4 μL of each 10 μM forward and reverse primers, and 4 μL of gDNA with nuclease-free water added to reach a final volume of 25 μL. *L. martiniquensis* DNA and *Trypanosoma evansi* DNA were used as positive controls, while sterile distilled water (template DNA-free water) was employed as a negative control. The PCR products were then subjected to electrophoresis using a 1.5% agarose gel and stained with ethidium bromide. The gels were then visualized using the GelDoc Go Imaging System and Image Lab software version 6.1 (Bio-Rad, California, USA). Positive PCR products were inserted into the pGEM® T-Easy vector (Promega, Wisconsin, USA), and plasmid extraction was performed and sent for sequencing by Macrogen, Inc. in Seoul, South Korea.

Identification of blood meal source by host-specific multiplex PCR

Amplification of mitochondrial cytochrome b (*cytb*) gene was used to detect the presence of vertebrate host-specific DNA in the engorged midges. Multiplex PCR was performed with primers specific to humans (Human741F), cattle (Cow121F), dog (Dog368F), pig (Pig573F), and chicken (Chick1123R) DNA and universal primer of mammal (UNREV1025) and chicken (UNFOR1029) [4, 5]. The multiplex PCR was performed under the following PCR conditions: denaturation at 95 °C for 5 min, then 35 cycles of 95 °C for 1 min, annealing at 58 °C for 1 min, and extension at 72 °C for 1 min, with a final extension at 72 °C for 7 min [6]. Extracted DNA from the blood of humans (*Homo sapiens*), cows (*Bos indicus*), dogs (*Canis lupus familiaris*), pigs (*Sus scrofa*), and chickens (*Gallus gallus*) was used as a positive control. Sterilized distilled water was used as a negative control (DNA-free water). All PCR amplification protocols were carried out using the Green Hot Start PCR Master Mix Direct-Load 2× (Biotectrabbit, Berlin, Germany) according to the recommended instructions. The multiplex PCR products were separated by 2% agarose gel electrophoresis and stained with ethidium bromide. The

successfully amplified products were demonstrated by 334, 562, 680, 453, and 95 bp for humans, cows, dogs, pigs, and chickens respectively. Alternative conventional PCR using UNFOR403 and UNREV1025 primers was performed to detect the presence of mammalian DNA in specimens that did not get amplified by multiplex PCR. The PCR conditions for mammal blood screening were identical to the multiplex PCR. Specimens that did not react in both the multiplex PCR and the mammal blood screening were regarded as negative.

Phylogenetic construction and species delimitation analysis of *Culicoides* biting midges

The *COI* nucleotide sequences obtained were subjected to editing and analysis using BioEdit Sequences Alignment Editor Version 7.2.5 [7]. The consensus nucleotide sequences were then compared with sequences available in the NCBI database using a Basic Local Alignment Search Tool (BLAST) (https://blast.ncbi.nlm.nih.gov/Blast.cgi) and the Barcode of Life Database (BOLD) (https://www.boldsystems.org/). The construction of the phylogenetic tree was achieved by employing the maximum likelihood (ML) method with 1000 bootstrap tests, utilizing the Molecular Evolutionary Genetics Analysis software, version 11 (MEGA11) [8]. The maximum likelihood (ML) tree was then visualized and edited using FigTree version 1.4.4 (https://tree.bio.ed.ac.uk/software/FigTree/). The intraspecific variations were calculated using the Kimura 2-parameter (K2P) model in MEGA11 [8].

For the species delimitation, the *COI* sequences of *Culicoides* species were identified at the Molecular Operational Taxonomic Unit (MOTU) level generated by the 3 algorithms: Assemble Species by Automatic Partitioning (ASAP) [9], Poisson Tree Processes (PTP) [10], and TCS haplotype network [11]. The ASAP method is a distance-based method implemented using a web server (https://bioinfo.mnhn.fr/abi/public/asap/). This method beneficially distinguishes between interspecific divergence and intraspecific variations. Analysis of ASAP was performed with default parameters and simple distances (*p*-distance), and the best partition was evaluated with the lowest ASAP score. The PTP method is tree-based and seeks to differentiate species' speciation. Analysis of PTP was assessed using the maximum likelihood phylogenetic tree as input, single rate method, and *p*-value = 0.001, in the web server (https://mptp.h-its.org/#/tree).

The haplotype network of *Culicoides* species was generated directly from the *COI* sequence alignment in DnaSP version 6 software (http://www.ub.edu/dnasp/) [12] and visualized using the parsimony network by the TCS method in Population Analysis with Reticulate Trees software (PopART) version 1.7 (https://popart.maths.otago.ac.nz/) [13]. The genetic diversity and neutrality test of *Leishmania* populations and other trypanosomatids were evaluated using the DnaSP v6 [12], and then the haplotype network based on the minimum spanning network was generated using PopART v1.7 [13].

References

1. Manomat J, Leelayoova S, Bualert L, Tan-Ariya P, Siripattanapipong S, Mungthin M, et al. Prevalence and risk factors associated with *Leishmania* infection in Trang Province, southern Thailand. PLoS Negl Trop Dis. 2017 Nov 20;11(11):e0006095. doi: 10.1371/journal.pntd.0006095. PMID: 29155831; PMCID: PMC5714378.

2. Noyes HA, Stevens JR, Teixeira M, Phelan J, Holz P. A nested PCR for the ssrRNA gene detects *Trypanosoma binneyi* in the platypus and *Trypanosoma* sp. in wombats and kangaroos in Australia. Int J Parasitol. 1999 Feb;29(2):331-9. doi: 10.1016/s0020-7519(98)00167-2. Erratum in: Int J Parasitol 2000 Feb;30(2):228. PMID: 10221634.

3. Srisuton P, Phumee A, Sunantaraporn S, Boonserm R, Sor-Suwan S, Brownell N, et al. Detection of *Leishmania* and *Trypanosoma* DNA in Field-Caught Sand Flies from Endemic and Non-Endemic Areas of Leishmaniasis in Southern Thailand. Insects. 2019 Aug 2;10(8):238. doi: 10.3390/insects10080238. PMID: 31382501; PMCID: PMC6722825.

4. Kent RJ, Norris DE. Identification of mammalian blood meals in mosquitoes by a multiplexed polymerase chain reaction targeting cytochrome B. Am J Trop Med Hyg. 2005 Aug;73(2):336-42. PMID: 16103600; PMCID: PMC4147110.

5. Kent RJ, Thuma PE, Mharakurwa S, Norris DE. Seasonality, blood feeding behavior, and transmission of *Plasmodium falciparum* by *Anopheles arabiensis* after an extended drought in southern Zambia. Am J Trop Med Hyg. 2007 Feb;76(2):267-74. PMID: 17297034; PMCID: PMC4152308.

6. Sunantaraporn S, Hortiwakul T, Kraivichian K, Siriyasatien P, Brownell N. Molecular Identification of host blood meals and detection of blood parasites in *Culicoides* latreille (Diptera: Ceratopogonidae) collected from Phatthalung province, southern Thailand. Insects. 2022 Oct 8;13(10):912. doi: 10.3390/insects13100912. PMID: 36292860; PMCID: PMC9604321.

7. Hall TA. BioEdit: a user-friendly biological sequence alignment editor and analysis program for windows 95/98/NT. Nucleic Acids Symp Ser. 1999;41:95–8.

8. Tamura K, Stecher G, Kumar S. MEGA11: molecular evolutionary genetics analysis version 11. Mol Biol Evol. 2021;38:3022–7. https://doi.org/10.1093/molbev/msab120.

9. Puillandre N, Brouillet S, Achaz G. ASAP: assemble species by automatic partitioning. Mol Ecol Resour. 2021 Feb;21(2):609-620. doi: 10.1111/1755-0998.13281. Epub 2020 Nov 20. PMID: 33058550.

10. Kapli P, Lutteropp S, Zhang J, Kobert K, Pavlidis P, Stamatakis A, et al. Multi-rate Poisson tree processes for single-locus species delimitation under maximum likelihood and Markov chain Monte Carlo. Bioinformatics. 2017 Jun 1;33(11):1630-1638. doi: 10.1093/bioinformatics/btx025. PMID: 28108445; PMCID: PMC5447239.

11. Clement M, Snell Q, Walke P, Posada D, Crandall K. TCS: estimating gene genealogies. Proc 16th Int Parallel Distrib Process Symp. 2002;2:184.

12. Rozas J, Ferrer-Mata A, Sánchez-DelBarrio JC, Guirao-Rico S, Librado P, Ramos-Onsins SE, et al. DnaSP 6: DNA sequence polymorphism analysis of large data sets. Mol Biol Evol. 2017;34:3299–302. https://doi.org/10.1093/molbev/msx248.

13. Leigh JW, Bryant D. PopART: Full-feature software for haplotype network construction. Methods Ecol Evol. 2015;6:1110–6.
