# Supplementary figures and images for: DNA barcoding of Culicoides biting midges (Diptera: Ceratopogonidae) and detection of Leishmania and other trypanosomatids in southern Thailand
Source: Parasit Vectors. 2025 May 29;18:194. doi: 10.1186/s13071-025-06812-0 (PMC12121006; doi:10.1186/s13071-025-06812-0)

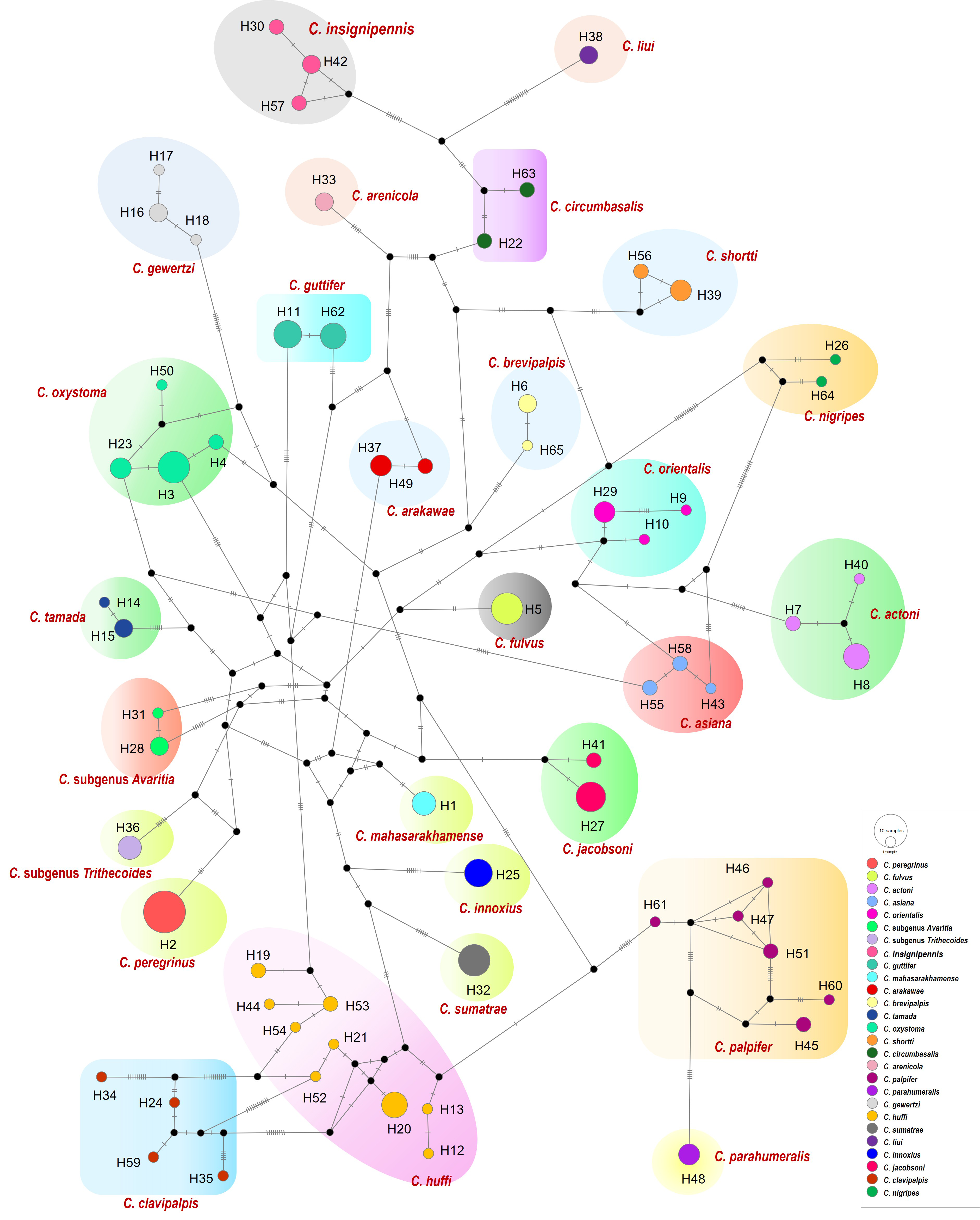

Supplement: Supplementary file 2 — Additional file 2: Figure S1. The TCS haplotype network of COI sequences of Culicoides biting midges from this study. [file 13071_2025_6812_MOESM2_ESM.tif]
