# Supplementary material for: DNA barcoding of Culicoides biting midges (Diptera: Ceratopogonidae) and detection of Leishmania and other trypanosomatids in southern Thailand
Source: Parasit Vectors. 2025 May 29;18:194. doi: 10.1186/s13071-025-06812-0 (PMC12121006; doi:10.1186/s13071-025-06812-0)
Supplement: Supplementary file 3 — Additional file 3: Table S1. Morphological identification and nucleotide search identified results in the BOLD and BLAST databases of Culicoides species collected from this study. [file 13071_2025_6812_MOESM3_ESM.pdf]

Table S1 Morphological identification and nucleotide search identified results in the BOLD and BLASTn databases of *Culicoides* species collected from this study

Morphological based identification	No. of sequences	BOLD Search		GenBank Search	
		Closest species	Average identity (%)	Closest species	Average identity (%)
C. actoni	6	*C. actoni* (GMMSO1324-18)	98.65	*C. actoni* (KT352618)	99.40
		Culicoides sp. (GMMST622-18)	99.07		
C. arakawae	2	*C. arakawae* (GBMNF24391-22)	96.76	*C. arakawae* (MZ189966)	99.56
		Culicoides sp. (GBAAW62226-24)	97.76		
C. arenicola	1	*C. arenicola* (CULIC1715-15)	99.77	*C. arenicola* (ON002391)	99.78
C. asiana	1	*C. asiana* (CUSCR549-11)	98.18	*C. asiana* (MW496167)	98.25
C. brevipalpis	1	*C. brevipalpis* (CUSCR2819-14)	99.00	*C. brevipalpis* (AB360998)	99.59
C. circumbasalis	1	*C. circumbasalis* (CUSCH750-17)	99.76	*C. circumbasalis* (ON002403)	99.76
C. clavipalpis	3	*C. clavipalpis* (CUSCH656-16)	95.20	*C. clavipalpis* (OR073919.1)	91.85
C. fulvus	6	*C. fulvus* (CUSCR2848-14)	99.44	*C. fulvus* (KT352206(100%), KT352643(100%))	99.63
C. gewertzi	3	*C. gewertzi* (GMMST1269-18)	99.35	*C. gewertzi* (ON002368)	99.15
		Culicoides sp. (CTISA5593-16)	95.59		
C. guttifer	7	*C. guttifer* (GBMND90502-21)	98.95	*C. guttifer* (MW496174)	99.23
		Culicoides sp. (GMBCN434-15)	99.06		
C. huffi	11	*C. huffi* (GMMNR773-18)	98.32	*C. huffi* (OP741195(100%), MW496213(100%), KY441772(100%))	99.37
		Culicoides sp. (GMBCE784-15)	94.89		
C. innoxius	5	*C. innoxius* (GMMNK189-18)	98.61	*C. innoxius* (OR073947)	100.00
C. insignipennis	5	*C. insignipennis* (CUYUN299-19)	97.70	*C. insignipennis* (PQ340919)	98.32
C. jacobsoni	7	*Culicoides* sp. (THAMD15852-22)	99.79	*C. jacobsoni* (MZ189959)	99.72
C. liui	1	*C. liui* (CULIC1702-15)	100.00	*C. liui* (ON002396)	99.34
C. mahasarakhamense	3	*C. mahasarakhamense* (GBMNF24438-22)	99.43	*C. mahasarakhamense* (MZ191855)	100.00
		Culicoides sp. (GBMND90586-21)	99.19		
C. nigripes	1	No match	-	*C. nigripes* (MZ189960)	94.10
C. orientalis	3	*C. orientalis* (THAMJ5897-23)	98.44	*C. orientalis* (MK760223)	98.91
C. oxystoma	11	*C. oxystoma* (GBAAW11134-24)	98.83	*C. oxystoma* (OQ536028)	99.23
C. palpifer	3	*C. palpifer* (GBMNF24498-22)	97.26	*C. palpifer* (MK917492)	97.70
		Culicoides sp. (GMPXA12458-23)	99.22		
C. parahumeralis	1	*C. parahumeralis* (THAMD22919-23)	97.70	*C. parahumeralis* (MH135786)	97.92
C. peregrinus	14	*C. peregrinus* (GBMND90656-21)	99.63	*C. peregrinus* (KY433458(100%), MW496289(100%), MK917543(100%))	99.96
C. shortti	3	*C. shortti* (GMMNT181-18)	99.35	*C. shortti* (MW496314)	99.49
C. subgenus *Avaritia*	4	No match	-	*C. fulvus* (KT352703)	88.67
C. subgenus *Trithecoides*	5	*Dasyhelea ludingensis* (GMTTB5990-23)	99.02	Ceratopogonidae sp. (KX051897)	97.92
		Forcipomyia (GMMSG591-18)	99.37		
C. sumatrae	7	*C. sumatrae* (THAMD23389-23)	99.65	*C. sumatrae* (MZ191875)	99.56
		Culicoides sp. (THAMI9167-23)	99.48		
C. tamada	3	*C. tamada* (THAMD8720-22)	98.57	*C. tamada* (ON002378)	99.30
		Culicoides sp. (GMBCG2385-15)	94.5		
