# Supplementary material for: DNA barcoding of Culicoides biting midges (Diptera: Ceratopogonidae) and detection of Leishmania and other trypanosomatids in southern Thailand
Source: Parasit Vectors. 2025 May 29;18:194. doi: 10.1186/s13071-025-06812-0 (PMC12121006; doi:10.1186/s13071-025-06812-0)
Supplement: Supplementary file 4 — Additional file 4: Table S2. K2P intraspecific genetic divergences of Culicoides species were collected in this study. [file 13071_2025_6812_MOESM4_ESM.pdf]

Table S2 K2P intraspecific genetic divergences of *Culicoides* species were collected in this study

Species	Number of sequences	K2P intraspecific divergence (min-max)
Culicoides actoni	6	0.0000-0.0477
Culicoides arakawae	2	0.0000
Culicoides arenicola	1	N/A
Culicoides asiana	1	N/A
Culicoides brevipalpis	1	N/A
Culicoides circumbasalis	1	N/A
Culicoides clavipalpis	3	0.1091-0.1880
Culicoides fulvus	6	0.0000-0.0037
Culicoides gewertzi	3	0.0074-0.0150
Culicoides guttifer	7	0.0000
Culicoides huffi	11	0-0.1837
Culicoides innoxius	5	0.0000
Culicoides insignipennis	5	0.0000-0.0349
Culicoides jacobsoni	7	0.0000-0.0149
Culicoides liui	1	N/A
Culicoides mahasarakhamense	3	0.0037-0.0074
Culicoides nigripes	1	N/A
Culicoides orientalis	3	0.0344-0.0814
Culicoides oxystoma	11	0.0037-0.0229
Culicoides palpifer	3	0.0111-0.0964
Culicoides parahumeralis	1	0.0000
Culicoides peregrinus	14	0.0000-0.0037
Culicoides shortti	3	0.0000
C. subgenus *Avaritia*	4	0.0000-0.0074
C. subgenus *Trithecoides*	5	0.0000-0.0037
Culicoides sumatrae	7	0.0000
Culicoides tamada	3	0.0000-0.0122
