# Supplementary material for: DNA barcoding of Culicoides biting midges (Diptera: Ceratopogonidae) and detection of Leishmania and other trypanosomatids in southern Thailand
Source: Parasit Vectors. 2025 May 29;18:194. doi: 10.1186/s13071-025-06812-0 (PMC12121006; doi:10.1186/s13071-025-06812-0)
Supplement: Supplementary file 5 — Additional file 5: Table S3. Culicoides species identification, number of positive specimens for Leishmania, and other trypanosomatids collected from four different areas in southern Thailand. [file 13071_2025_6812_MOESM5_ESM.pdf]

Table S3 *Culicoides* species identification, number of positive specimens for *Leishmania,* and other trypanosomatids collected from four different areas in southern Thailand

Provinces	*Culicoides* species	No. of tested	Positive trypanosomatids detection			
			ITS-PCR	*SSU* rRNA-PCR	Prevalence rate (95% CI)	
			Leishmania	Other trypanosomatids	*Leishmania*	Other trypanosomatids
Ron Phibun Nakhon Si Thammarat (NST1) (n=334)	*C. actoni*	8	0	0	0	0
	C. arakawae	2	0	0	0	0
	C. asiana	1	0	0	0	0
	C. fulvus	4	0	0	0	0
	C. guttifer	11	0	0	0	0
	C. huffi	13	0	0	0	0
	C. innoxius	20	**1**	0	5.00 (0.00-25.41)	0
	C. insignipennis	19	0	0	0	0
	C. jacobsoni	12	0	0	0	0
	C. liui	1	0	0	0	0
	C. oxystoma	79	**3**	0	3.80 (0.00-11.03)	0
	C. peregrinus	138	**27**	**11**	19.57 (13.76-27.02)	7.97 (4.37-13.84)
	C. shortti	3	0	0	0	0
	C. sumatrae	12	0	0	0	0
	C. palpifer	3	0	0	0	0
	C. parahumeralis	1	0	0	0	0
	C. subgenus *Trithecoides*	7	0	0	0	0
Sichon Nakhon Si Thammarat (NST2) (n=68)	*C. arenicola*	1	0	0	0	0
	C. calvipalpis	4	0	0	0	0
	C. subgenus *Avaritia*	5	**1**	0	20.00 (2.03-64.04)	0
	C. guttifer	10	**2**	0	20.00 (4.59-52.06)	0
	C. huffi	11	**2**	0	18.18 (3.99-48.85)	0
	C. innoxius	3	0	0	0	0
	C. insignipennis	3	0	0	0	0
	C. jacobsoni	4	0	0	0	0
	C. orientalis	4	**1**	0	25.00 (3.41-71.09)	0
	C. oxystoma	1	0	0	0	0
	C. peregrinus	2	0	0	0	0
	C. sumatrae	1	0	0	0	0
	C. subgenus *Trithecoides*	19	0	0	0	0
Phunphin Surat Thani (ST) (n=97)	*C. calvipalpis*	1	0	0	0	0
	C. guttifer	5	**1**	0	20.00 (2.03-64.04)	0
	C. huffi	70	**6**	0	8.57 (3.67-17.78)	0
	C. innoxius	1	**1**	0	100 (16.75-100)	0
	C. mahasarakhamense	1	0	0	0	0
	C. nigripes	1	**1**	0	100 (16.75-100)	0
	C. oxystoma	13	0	0	0	0
	C. peregrinus	3	0	0	0	0
	C. subgenus *Trithecoides*	1	0	0	0	0
	C. circumbasilis	1	0	0	0	0
Khlong Thom Krabi (KB) (n=217)	*C. actoni*	4	0	0	0	0
	C. brevipalpis	3	0	0	0	0
	C. fulvus	7	0	0	0	0
	C. orentalis	15	0	0	0	0
	C. huffi	48	0	0	0	0
	C. peregrinus	61	0	0	0	0
	C. guttifer	64	0	0	0	0
	C. mahasarakhamense	2	0	0	0	0
	C. oxystoma	5	0	0	0	0
	C. gewertizi	3	0	0	0	0
	C. tamada	3	0	0	0	0
	C. subgenus *Trithecoides*	2	0	**2**	0	100 (29.02-100)
	**Total**	716	**46**	**13**	6.42 (4.84-8.48)	1.82 (1.03-3.11)
