# Supplementary material for: DNA barcoding of Culicoides biting midges (Diptera: Ceratopogonidae) and detection of Leishmania and other trypanosomatids in southern Thailand
Source: Parasit Vectors. 2025 May 29;18:194. doi: 10.1186/s13071-025-06812-0 (PMC12121006; doi:10.1186/s13071-025-06812-0)
Supplement: Supplementary file 7 — Additional file 7: Table S5. Host blood meal identification of Culicoides biting midges collected from Ron Phibun district in Nakhon Si Thammarat, southern Thailand. [file 13071_2025_6812_MOESM7_ESM.pdf]

Table S5. Host blood meal identification of *Culicoides* biting midges collected from Ron Phibun district in Nakhon Si Thammarat, southern Thailand

Species	Number of blood-fed females	Single blood meal (*n*)					Multiple blood meals (*n*)
		Human	Cow	Dog	Pig	Chicken	
C. actoni	2	0	2	0	0	0	-
C. fulvus	3	0	3	0	0	0	-
C. huffi	1	0	0	0	0	1	-
C. innoxius	25	0	24	0	0	0	Cow and chicken (1)
C. insignipennis	18	0	18	0	0	0	-
C. jacobsoni	14	0	14	0	0	0	-
C. orientalis	12	0	12	0	0	0	Human and cow (1)
C. oxystoma	5	0	4	0	0	0	Human and cow (1)
C. peregrinus	10	0	8	0	0	0	-
C. shortti	1	0	1	0	0	0	-
C. sumatrae	12	0	11	0	0	0	Human and cow (1)
C. subgenus *Trithecoides*	56	0	53	3	0	0	-
**Total**	**159**	**0**	**149**	**3**	**0**	**1**	**4**
